# Characterization of Bacteria Associated with Pinewood Nematode *Bursaphelenchus xylophilus*

**DOI:** 10.1371/journal.pone.0046661

**Published:** 2012-10-16

**Authors:** Claudia S. L. Vicente, Francisco Nascimento, Margarida Espada, Pedro Barbosa, Manuel Mota, Bernard R. Glick, Solange Oliveira

**Affiliations:** 1 NemaLab, Instituto de Ciências Agrárias e Ambientais Mediterrânicas (ICAAM), Universidade de Évora, Évora, Portugal; 2 Laboratório de Microbiologia do Solo, Instituto de Ciências Agrárias e Ambientais Mediterrânicas (ICAAM), Universidade de Évora, Évora, Portugal; 3 Department of Biology, University of Waterloo, Waterloo, Ontario, Canada; University of the West of England, United Kingdom

## Abstract

Pine wilt disease (PWD) is a complex disease integrating three major agents: the pathogenic agent, the pinewood nematode *Bursaphelenchus xylophilus*; the insect-vector *Monochamus* spp.; and the host pine tree, *Pinus* sp. Since the early 80's, the notion that another pathogenic agent, namely bacteria, may play a role in PWD has been gaining traction, however the role of bacteria in PWD is still unknown. The present work supports the possibility that some *B. xylophilus*-associated bacteria may play a significant role in the development of this disease. This is inferred as a consequence of: (i) the phenotypic characterization of a collection of 35 isolates of *B. xylophilus*-associated bacteria, in different tests broadly used to test plant pathogenic and plant growth promoting bacteria, and (ii) greenhouse experiments that infer the pathogenicity of these bacteria in maritime pine, *Pinus pinaster*. The results illustrate the presence of a heterogeneous microbial community associated with *B. xylophilus* and the traits exhibited by at least, some of these bacteria, appear to be related to PWD symptoms. The inoculation of four specific *B. xylophilus*-associated bacteria isolates in *P. pinaster* seedlings resulted in the development of some PWD symptoms suggesting that these bacteria likely play an active role with *B. xylophilus* in PWD.

## Introduction

Pine wilt disease (PWD) is a worldwide threat to forests, which has severely affected both Asian (Japan, China, Taiwan and South Korea) and European (Portugal and Spain) countries causing huge and irreversible economic and environmental damage [1], [2]. The pathogenic agent of PWD, *Bursaphelenchus xylophilus*, known as the pinewood nematode (PWN), is a migratory plant parasitic nematode that infects different species of pine trees (*Pinus* spp.), causing mainly blockage of the host vascular vessels and cavitation, thereby leading to development of wilting symptoms [3]. The PWN, vectored by a pine sawyer beetle, *Monochamus* spp. [4], [5], has been characterized by its association with diverse bacterial communities that have been investigated as potential “helpers” in the complex system of PWD [6]. The hypothesis of a mutualistic relationship between these bacteria and the PWN is supported by a number of different factors including: (i) non-development of PWD in *Pinus thunbergii* seedlings inoculated with aseptic PWN, in contrast with the results of joint inoculation of aseptic PWN plus bacteria [7], [8]; (ii) *Pseudomonas fluorescens* GcM5-1A, isolated from PWN, significantly increasing the fecundity and reproduction rate of the PWN [9]; and (iii) the population dynamics of bacteria and PWN during the different stages of PWD [10], [11]. Interestingly, different microbial communities have been found to be associated with the PWN in different countries. In the Asian forestlands affected by PWD, the genus *Bacillus* in Japan [12]; the genus *Pseudomonas* in China [8]; and the genera *Brevibacterium, Burkholderia*, *Enterobacte,r Ewingella*, and *Serratia* in Korea, were the most representative bacteria associated with the PWN [13]. In Europe, specifically in Portugal, the genera *Burkholderia* and *Pseudomonas* [14], as well as bacteria from the Enterobacteriaceae family were found associated with *B. xylophilus* [15]. Despite all efforts to better understand the nematode-bacterial relationship, the contribution of these microbial communities in the development of PWD is not well understood. In this work, a collection of bacteria isolated from *B. xylophilus* [15] were phenotypically characterized in terms of some of the mechanisms used by plant pathogenic and plant growth-promoting bacteria. In addition, the pathogenicity of these bacteria in *Pinus pinaster* seedlings was assessed.

## Materials and Methods

### Bacterial isolation

Thirty-five bacterial isolates (Table 1), representative of the main bacterial genera isolated from *Bursaphelenchus xylophilus*, were used in this study. Two sampling sources of portuguese *B. xylophilus* were previously screened for bacteria: laboratory culture nematodes, obtained from symptomatic pine trees in Portugal and maintained in barley cultures for more than three years (LCN), and nematodes directly obtained from symptomatic pine trees collected in PWD affected area in Portugal (PWN) [15].

**Table 1:** Traits of various bacteria associated with *Bursaphelenchus xylophilus*.

Isolate identifier	16S rRNA identification	Antibiotic					Cellulase activity	ACC deaminase	Biofilm formation	Phosphate solubilization	Siderophore production	Hypersensitive Reaction
		Amp	Ery	Kan	Tet	Rif						
**LCN-4**	*Serratia sp.*	+	+	−	−	−	−	−	+	−	+++	+
**LCN-5**	*Stenotrophomonas sp.*	+	+	+	+	−	+	−	+	−	++	−
**LCN-16**	*Serratia sp.*	+	+	−	−	−	−	−	−	−	++	−
**LCN-23**	*Serratia sp.*	−	+	−	−	−	−	−	+	−	++	−
**LCN-25**	*Enterobacter sp.*	+	+	−	−	+	−	−	−	−	++	+
**LCN-27**	*Enterobacter sp.*	+	+	−	+	+	−	−	+	−	+	−
**LCN-36**	*Enterobacter sp.*	+	+	−	−	+	+	−	+	−	+	−
**LCN-37**	*Enterobacter sp.*	+	+	−	+	+	−	−	+	−	++	−
**LCN-45**	*Serratia sp.*	+	+	−	+	+	−	−	−	−	++	−
**LCN-46**	*Klebsiella sp.*	+	+	−	−	+	+	−	−	−	+	+
**LCN-49**	*Enterobacter sp.*	−	+	+	+	+	−	−	+	−	−	−
**LCN-56**	*Stenotrophomonas sp.*	+	+	+	+	+	−	−	+	+	−	−
**LCN-58**	*Stenotrophomonas sp.*	+	+	+	+	+	+	−	+	−	−	−
**LCN-69**	*Pseudomonas sp.*	+	+	−	−	+	−	−	+	−	++	+
**LCN-71**	*Acinetobacter sp.*	+	+	−	+	+	+	−	+	−	++	−
**LCN-72**	*Acinetobacter sp.*	+	+	−	−	+	+	−	−	−	+	−
**LCN-87**	*Pseudomonas sp.*	+	+	+	−	−	+	−	−	−	+++	−
**PWN-89**	*Rahnella sp.*	+	+	−	+	+	−	−	+	−	+++	+
**PWN-98**	*Serratia sp.*	+	+	+	+	+	−	−	+	−	+	−
**PWN-99**	*Serratia sp.*	+	+	−	−	+	+++	−	+	−	+++	−
**PWN-116**	*Ewingella sp.*	−	+	−	−	−	−	−	+	−	++	−
**PWN-119**	*Erwinia sp.*	+	+	−	+	+	+	−	+	−	++	+
**PWN-120**	*Ewingella sp.*	+	+	−	+	+	+	−	+	−	++	+
**PWN-128**	*Serratia sp.*	+	+	−	+	+	+	−	+	−	++	+
**PWN-129**	*Serratia sp.*	+	+	−	+	+	−	−	+	−	++	−
**PWN-136**	*Burkholderia sp.*	+	+	−	+	+	−	−	+	−	++	−
**PWN-142**	*Serratia sp.*	+	+	+	+	+	−	−	+	−	+	−
**PWN-144**	*Rahnella sp.*	+	+	+	+	+	+	−	+	+	+	−
**PWN-146**	*Serratia sp.*	+	+	+	+	+	+	−	+	−	+	+
**PWN-153**	*Rahnella sp.*	+	+	−	+	+	+	−	+	+	+	−
**PWN-183**	*Pantoea sp.*	+	+	−	+	+	−	−	+	−	+	−
**PWN-186**	*Serratia sp.*	+	+	+−	+	+	−	−	+	−	+	−
**PWN-196**	*Erwinia sp.*	+	+	+	+	+	−	−	+	−	++	−
**PWN-212**	*Rahnella sp.*	+	+	+	+	+	−	−	+	−	++	−
**PWN-228A**	*Kocuria sp.*	+	+	+	+	+−	++	−	+	−	−	−

Qualitative scale: (−), no production/activity; (+), poor-production/activity; (++), moderate-production/activity; and (+++) high-production/activity. For antibiotic resistance: (−) indicates non-resistant and (+) resistant. The bacterial isolates with positive HR in *Nicotiana tabacum* are indicated by (+).

### Antibiotic sensitivity/resistance

Bacterial sensitivity/resistance to five different antibiotics, namely ampicillin (Amp) (50 µg/ml), erythromycin (Ery) (50 µg/ml), kanamycin (Kan) (50 µg/ml), rifampicin (Rif) (50 µg/ml) and tetracycline (Tet) (15 µg/ml) was tested. The antibiotics were added separately to 5 ml TSB (tryptic soy broth, Merck®), before 20 µl of an overnight test culture was added. Bacterial cultures were incubated at 30°C in a shaking water bath at 200 rpm for 24–48 h. Sensitivity/resistance was determined based on the growth of the bacterial strains in the medium containing the different antibiotics.

### Cellulase activity

For each bacterial isolate, CMC (carboxymethylcellulose) plates [16] were spot-inoculated (5 µl) with overnight-culture, and incubated at 28°C for 48 h. Cellulase activity was observed by the formation of a zone of clearance around the bacterial colony, after flooding the CMC plates with Gram's iodine solution (2.0 g KI and 1.0 g iodine in 300 ml distilled water) for 5 minutes. The production of cellulase was determined by comparing the diameter of the zone of clearance between the isolates. Testing for cellulase activity was repeated three times for each bacterial isolate.

### ACC (1-aminocyclopropane-1-carboxylate) deaminase activity

Bacterial cultures were grown overnight in TSB medium, centrifuged at 10000 *g* and washed twice with DF minimal medium [17], before resuspended in DF minimal medium with an ACC final concentration of 5 mM as the sole source of nitrogen. Cells were then incubated for approximately 40 h at 30°C and 200 rpm. After induction, ACC deaminase activity was measured based on the determination of α-ketobutyrate resulting from ACC cleavage by ACC deaminase, as described by Penrose and Glick [18].

### Biofilm formation

Qualitative evaluation of biofilm formation was conducted according to Koczan et al. [19]. A 5 µl aliquot of cells grown overnight in TSB at 28°C was inoculated into 125 µl of sterile LB medium (Luria broth, Merck®) in individual wells of a 96-well PVC plate (each receiving one bacterial isolate), and incubated for 16 h at 28°C with minimal agitation. Following bacterial growth, the medium was discarded and the wells were filled with 10% crystal violet stain. After 1 h at room temperature, the crystal violet was decanted and the wells were gently rinsed with water. The presence of crystal violet-stained rings was determined visually. Biofilm formation assessment was repeated three times.

### Phosphate solubilization

Bacterial phosphate solubilization activity was screened according to Freitas et al. [20] in PDYA-CaP medium (potato-dextrose yeast extract agar supplemented with calcium phosphate CaHPO_4_). For each isolate, three plates of PDYA-CaP were spot- inoculated with 5 µl of overnight culture in TSB medium, and incubated at 28°C for 14 days. This procedure was three times repeated for each bacterial isolate. Phosphate solubilization activity determined by measuring the clearance zone (area of solubilization) developed around the colony and comparison of this zone with the colony diameter. The phosphate solubilization index = (colony+clearance zone diameter)/colony diameter). The cut off value of phosphate solubilization index is 1, indicating the bacterial isolate has not the ability to solubilize phosphate.

### Siderophore production

Siderophore production was qualitatively measured based on the method described by Schwyn and Neilands [21]. Siderophores produced by bacteria take up iron from a complex with the dye, chrome azurol S (CAS), and a positive reaction is indicated by a color change of the CAS reagent from blue to orange. About 5 µl of an overnight bacterial culture grown in King's B medium [22] was spotted onto a CAS agar plate [23] and incubated at 30°C for 2 days. Siderophore production was evaluated three times for each *B. xylophilus* associated bacteria.

### Exopolysaccharides production

Screening and quantitative evaluation of exopolysaccharides (EPS) production was performed as described by Geel-Schutten et al. [24] using glucose as a carbon source. Modified MRS medium supplemented with glucose (100 g/l) [24] was inoculated with overnight cultures, and incubated for 3 days at 28°C. After incubation, 1 ml of the culture was sampled and centrifuged at 11000 *g* for 4 minutes. For 1 ml of supernatant for each sample, two volumes of cold absolute ethanol was added and stored overnight at 4°C. The chilled mixture was subsequently centrifuged (15 minutes at 2000 *g*), and the pellet that formed was resuspended in 1 ml distilled water. The EPS was reprecipitated with two volumes of cold absolute ethanol, centrifuged and then the pellet was dried at 55°C for 3 days. EPS production was determined by weighing the dried precipitates. This entire experiment was repeated three times.

### IAA (Indole-3-acetic acid) production

Bacterial IAA production ability was measured following a minor variant of the method described by Glickmann and Dessaux [25], Patten and Glick [26] and Rashid et al. [27]. An aliquot of 20 µl of an overnight grown bacterial culture was used to inoculate 5 ml TSB medium containing tryptophan (500 µg/ml). A control was performed without tryptophan added to the medium. Bacterial cultures were incubated at 30°C for 24 h. Subsequently, cultures were centrifuged and 1 ml supernatant was mixed with 4 ml Salkowski's reagent [28], incubated for 20 min at room temperature before the absorbance was read (OD_535_). The concentration of IAA in each sample was calculated based on a standard curve ranging from 0.5 to 25 µg/ml IAA (Sigma). This procedure was repeated three times for each bacterial isolate.

### Gnotobiotic Root Elongation

The modified gnotobiotic root elongation assay was based upon the procedure described by Penrose and Glick [18]. Each bacterial isolate was prepared as follows: overnight cultures were centrifuged at 8000 *g* for 10 minutes at 4°C, the cell pellet was washed twice, and diluted with 0.03M MgSO_4_ to a final OD_600_ of 0.5. Canola seeds (*Brassica campestris* L.), previously surface sterilized with 70% (v/v) ethanol (1 minute) and 1% (v/v) sodium hypochlorite (10 minutes), were soaked in the bacterial inoculum for 1 hour. After incubation, 15 pre-treated seeds were sown in steam sterilized plant substrate SIRO® (N, 150–250 mg/l; P2O5, 150–250 mg/l; K, 300–500 mg/l), at a depth of 1 cm, and grown in a growth chamber under controlled conditions (24±1°C, 80% humidity; 12 h day/night photoperiod). Root elongation was measured five days after sowing. The trial consisted of two pots (12×12.5 cm) with 15 seeds each per treatment, distributed in a randomized block design.

### Hypersensitivity Reaction (HR)

HR tests were based on the procedure described by Umesha et al. [29]. Cell pellets from overnight bacterial cultures grown in TSB medium were washed three times in PBS (phosphate buffered saline) by centrifugation at 4000 *g* for 10 minutes. The optical density was adjusted to 0.5 (OD_600_). An aliquot of 200 µl of each bacterial suspension was infiltrated into the lower surface of a mature *Nicotiana tabacum* (tobacco) leaf by syringe (1 ml) injection, pressing the suspension against the leaf. The HR assay was repeated three times for each *B. xylophilus* associated bacteria. Tobacco plants were maintained under controlled growth chamber conditions (average temperature of 24°±2°C, 80% humidity, and a 14 h daylight photoperiod) for four days, when HR symptoms became visible (necrosis, yellowing of infiltrated area and leaf senescence). The type-strain, *Pseudomonas syringae* pv. *syringae* CPBR 314, was used as HR positive control. For negative control treatment, PBS was used.

### Pathogenicity tests in Pinus pinaster

#### Nematodes Rearing and Collection

The *B. xylophilus* isolate HF, collected in Setúbal region (SW Portugal) and maintained in the Nematology lab (University of Évora, Évora, Portugal), was employed in pathogenicity tests. Existing individuals were placed in Erlenmeyer flasks with *Botrytis cinerea*, cultured for a week in autoclaved barley/water medium and reared during 7 days at 25°C in darkness [30]. Nematodes were separated from the culture after 24 h in a Baermann tray, followed by Baermann funnel during 6 h. A suspension of about 750 nematodes (mixed-stages) was prepared in distilled water for inoculation.

#### Inoculation of *P. pinaster*

The pathogenicity tests in clone seedlings of one-year-old *Pinus pinaster* were carried out in glasshouse conditions (average temperature of 23°±2°C, 70–80% humidity) at the Instituto Nacional de Recursos Biológicos (Oeiras, Portugal). The inoculation procedure was conducted using the method of Futai and Furuno [31]. A small wound (3–5 mm) was made in the lower part of the pine stem, using a sterile blade. A sterilized piece of cotton was placed at the wound site and fixed there with Parafilm™ (inoculation point). Four bacterial isolates (selected by their phenotypic features, gnotobiotic root elongation and HR tests) were prepared as described above for HR tests, in a concentration of 10^7^–10^8^ CFU/ml. In addition, two control treatments were established: (i) a positive control with *B. xylophilus* inoculation ; and (ii) a negative control using sterile PBS. The experimental design consisted of 6 pine seedlings for each treatment randomly distributed. The trial was maintained for 45 days. At harvest time, a symptomology score [32] for each tree seedling was given as follows: 0, no needle discoloration; 1, only needles around the inoculation site were yellowish, needles in the other parts of the seedlings were green; 2: needles in the upper and lower part of the inoculation spot were brown yellowish, and needles in the top of the tree were grayish green; 3: needles in the upper and lower part of the inoculation spot were brown yellowish, and needles in the top were yellowish green; 4: all needles of the plant were yellowish brown; 5: all needles were brown. The disease incidence was calculated according to Fang [33]:
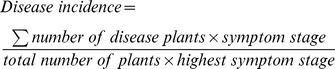



### Statistical analysis

Analysis of quantitative data (IAA and EPS production; canola root growth; and co-inoculation trials) was performed using the STATISTICA software version 7. One-way ANOVA analysis was conducted to infer the effect of *B. xylophilus* associated bacteria inoculation on canola root growth; the data was also analyzed using p*ost hoc* Tukey's test (*p*<0.05) for multiple mean comparisons.

## Results

### Bacterial characterization

**Figure 1:**
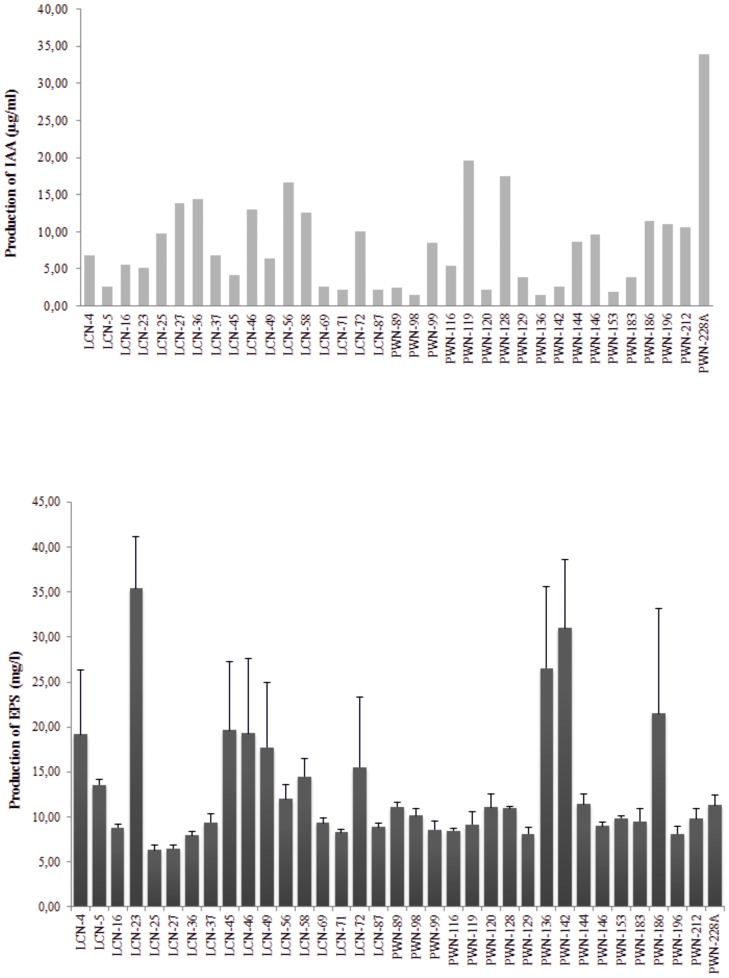
Characterization of bacteria associated with *Bursaphelenchus xylophilus* in terms of production of IAA (µg/ml) (a) and EPS (mg/l) (b).

The characterization of the 35 bacterial isolates obtained from a different nematode source (nematode from symptomatic *Pinus pinaster*, PWN; and laboratory cultured nematode, LCN) [15] were tested in terms of antibiotic resistance, cellulase and ACC deaminase activity, phosphate solubilization, and biofilm, EPS, IAA and siderosphore production (Table 1 and Fig. 1a and 1b). Five different antibiotics (Amp, Ery, Kan, Tet, and Rif) were tested at concentrations of 15 and 50 µg/ml. With the exception of Ery, to which all of the isolates were resistant, isolates were generally more sensitive, in a rank order, to Kan<Tet<Rif<Amp. Moreover, six isolates (*Erwinia sp.* PWN-196, *Rahnella sp.* PWN-212, *Serratia sp.* PWN-142, *Rahnella sp.* PWN-144, *Serratia sp.* PWN-98, and *Serratia sp.* PWN-146) obtained from nematodes of symptomatic trees, were resistant to all five antibiotics.

Nearly 46% of bacterial isolates tested were positive for cellulase activity, with *Kocuria sp.* PWN-228A and *Serratia sp.* PWN-99 demonstrating a moderate to high level of activity (Table 1). In contrast, ACC deaminase activity was not detected in any of the isolates. All bacteria produced IAA (Fig. 1a) in a range of 1.41 to 17.47 µg/ml. The highest production was recorded for *Kocuria sp.* PWN-228A with 33.96 µg/ml of IAA. In terms of EPS production (Fig. 1b), *Enterobacter sp.* LCN-23 displayed the highest level (35.4 mg/l), followed by *Serratia* sp. PWN-142 (31.0 mg/l) and *Burkholderia sp.* PWN-136 (26.5 mg/l). Nearly 40% of the isolates were moderate EPS-producers, ranging from 10.0 to 20.0 mg/ml of soluble sugars, and 49% of isolates were poor producers, yielding less than 10.0 mg/ml of soluble sugars on this test. Approximately 85% of the bacterial isolates were positive for biofilm formation on an inert surface. Phosphate solubilization was only detected in *Stenotrophomonas sp.* LCN-56, with an index of 1.40, and *Rahnella sp.* PWN-144 and *Rahnella sp.* PWN-153, both with an index of 1.17. Almost all the isolates were positive for the production of siderophores. A wide range of different levels of siderophore production was observed. *Serratia sp.* PWN-99 and *Rahnella sp.* PWN-89 were found to be substantial producers and, by contrast, *Enterobacter sp.* LCN-36 and *Klebsiella sp.* LCN-46 were poor siderophore producers.

**Figure 2:**
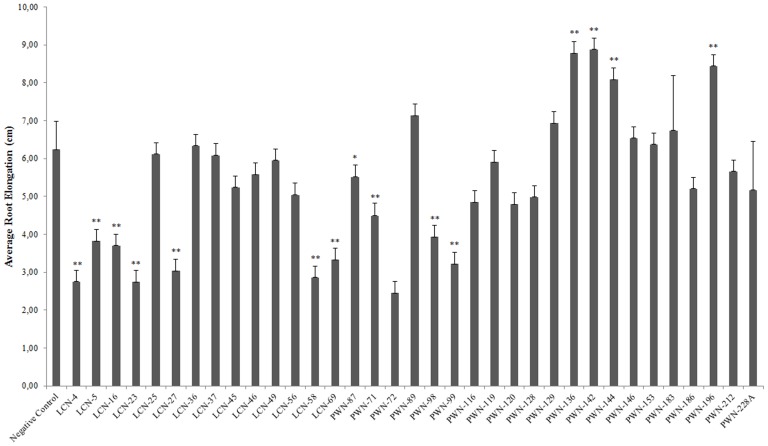
Gnotobiotic root elongation of *Brassica campestris* L. after inoculation of bacteria associated with *Bursaphelenchus xylophilus*. Statistical differences between *B. xylophilus* associated bacteria and the control treatment are noted by an asterisk (*P<0.05, **P<0.01, Tukey's test).

Approximately one third of the isolates had no significant effect on gnotobiotic root elongation in canola (Fig. 2). Significant inhibition of canola root growth in canola was observed with 11 bacterial isolates. For example, *Acinetobacter sp.* PWN-72 and *Serratia sp.* LCN-4 decreased canola root growth by 56 and 61%, respectively in comparison with the control treatment. Only four isolates (*Burkholderia sp.* PWN-136, *Serratia sp.* PWN-142, *Rahnella sp.* PWN-144 and *Erwinia sp.* PWN-196) induced a significant increase in canola root growth.

Of the 35 isolates, only nine bacterial isolates, and the plant pathogenic bacteria *Pseudomonas syringae* pv. *syringae* CPBR314, induced visible HR responses in tobacco leaves (typically cell necrosis around the inoculation point); these strains were *Ewingella* sp. PWN-120, *Enterobacter* sp. LCN-25, *Erwinia* sp. PWN-119, *Serratia sp.* PWN-128, *Klebsiella sp.* LCN-46, *Serratia sp.* PWN-146, *Pseudomonas sp.* LCN-69, *Serratia* sp. LCN-4 and *Rahnella* sp. PWN-89 (Table 1).

### Pathogenicity tests in *Pinus pinaster*

**Figure 3:**
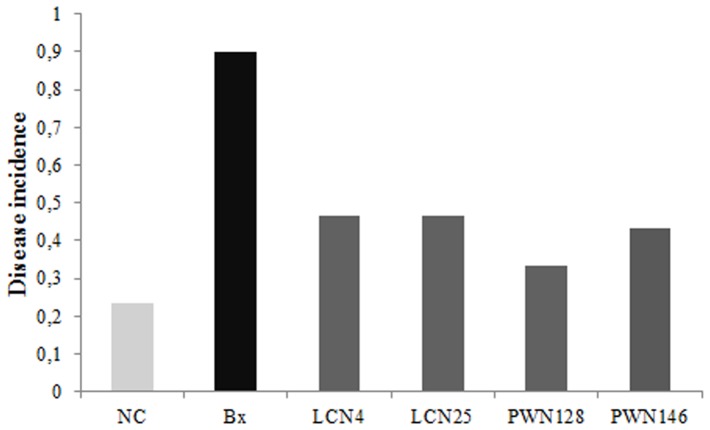
Disease incidence in *Pinus pinaster* after inoculation with *B. xylophilus* associated bacteria. NC, negative control treatment. Bx, *B. xylophilus*.

The selection of four bacterial isolates for the pathogenicity tests was based mostly on the results of canola root elongation and HR trials. The selected isolates were HR positive. Among laboratory culture nematodes, *Serratia* sp. LCN-4 and *Enterobacter* sp. LCN-25 were selected. *Serratia* sp. LCN-4 is a copious siderophore producer and significantly decreased canola root growth. *Serratia* LCN-25 produced severe HR in tobacco plants, is a moderate siderophore producer and did not affect canola root elongation. Among *B. xylophilus* from symptomatic pines, *Pantoea* sp. PWN-128 and *Serratia* sp. PWN-146 were selected. *Pantoea* sp. PWN-128 is resistant to four different antibiotics (Amp/Ery/Tet/Rif), a cellulase producer, forms biofilms on inert surfaces, is a moderate siderophore producer, is a relatively high IAA producer, and did not affect canola root elongation. *Serratia* sp. PWN-146 is resistant to all five antibiotics tested (Amp/Ery/Kan/Tet/Rif), a cellulase producer, forms biofilms, and did not affect canola root elongation. Figure 3 presents the disease incidence values for single inoculation of the isolates in one-year-old *P. pinaster*. The inoculation with the selected isolates resulted in the development of PWD symptoms, though with less severity than seedlings inoculated with *B. xylophilus*. The average symptom recorded was needle discoloration in the upper part of the inoculation spot. The disease incidence of *Serratia* sp. LCN-4 and *Enterobacter* sp. LCN-25 were the highest, followed by *Serratia* sp. PWN-146 and *Pantoea* sp. PWN-128. No disease symptoms were recorded in negative control *P. pinaster* seedlings.

## Discussion

The work reported here was undertaken in an effort to gain some insight into the role of *Bursaphelenchus xylophilus* associated bacteria in PWD. This effort included the phenotypic characterization of 35 bacteria isolated from *B. xylophilus*, using different tests broadly applied in the description of plant pathogenic and plant growth-promoting bacteria, as well as pathogenicity trials with the host tree *Pinus pinaster*. The results indicate the presence of a diverse microbial community associated with *B. xylophilus* including some strains with traits that may be related to PWD symptoms and *B. xylophilus* pathogenicity. Moreover, *B. xylophilus* associated bacteria isolated from the different nematode sources, namely laboratory cultures (LCN) and symptomatic pine (PWN), share common features such as moderate to high EPS/IAA and siderophore production, low phosphate solubilization ability, and absence of ACC deaminase activity. In contrast, most? PWN-bacteria isolates have shown to be more resistant to antibiotics, with moderate to high cellulolytic activity and able to form biofilm. The major differences between PWN- and LCN- bacteria may result from environment adaptation. The preliminary pathogenicity trial suggest that, at least in some cases, bacterial isolates inoculation alone, PWN- or LCN- bacteria, can lead to the development of PWD symptoms and, if in concert with the pathogenic agent *B. xylophilus*, these bacterial strains may increase the severity of those symptoms.

Many of the characteristics studied in this work may have dual functions in bacteria, and depending on the ecological niche or specific host, they can be perceived as either saprophytic features or as true pathogenic actions [34]. Microbial cellulases may be considered to be plant cell wall degrading enzymes including endo-β-1,4-glucanases, β-glucosidases and cellobiohydrolases [35]. Cellulolytic activity is present among many phytopathogens, although it is not a determining factor for pathogenicity, commonly found in saprophytes [35]. In the pathogen-system of PWD, cellulases are important *B. xylophilus* elicitors produced in the esophageal gland cells and are secreted through the stylet into the plant tissues, allowing progression of the nematode inside the host [36]. In this sense, it is reasonable to consider that cellulase production by associated bacteria may act as an adjunct in nematode infectivity. Among the collection of bacterial strains tested, we found that bacterial isolates from both sampling sources, laboratory culture nematodes (LCN) and symptomatic pine trees (PWN), had the ability to degrade cellulose, although this is not a feature common to all 35 strains.

The formation of biofilms is correlated with the production of EPS [37]. Biofilms are described as structured communities of microbial cells, of inter/intraspecies nature, surrounded by extracellular products that allow adhesion to living or non-living surfaces and provide a localized homeostatic environment [38], [39]. Numerous plant pathogenic bacteria that produce EPS and biofilm, the latter to a lesser extent, are believed to be responsible for wilting diseases such as in the case of xylem-colonizing bacteria *Ralstonia solanacearum*, *Pantoea stewartii* and *Xyllela fastidiosa* [40]. Although not all formed biofilms on an inert surface, all tested isolates were able to produce EPS, which may lead to the wilting symptoms of PWD.

Under iron stressed conditions, bacteria and fungi are able to produce low-molecular weight ferric ion specific chelating agents known as siderophores [41]. This ability is widespread in soil bacteria, however the role of these compounds in pathogenesis may be pathosystem-specific [42]. In this regard, siderophore-deficients mutant of *Ralstonia solanacearum* maintained their virulence [43] while *Erwinia chrysanthemi* was found to be dependent on siderophore production for systemic progression of maceration symptoms in the host [44]. In addition, Niño-Liu et al. [45] showed that siderophores play an important role in the pathogenesis of the xylem-colonizing bacterium *Xanthomonas oryzae* pv. *oryzae*. Almost all bacterial isolates examined in this study were siderophore-producers.

IAA functions in plants in diverse ways, varying from pathogenesis to phytostimulation, depending on the particular plant and its stage of growth, as well as the presence of stress factors. The IAA biosynthetic pathway of *Pseudomonas syringae* pv. *savastanoi* may be considered to be an intrinsic factor of its virulence, allowing the suppression the HR of host plants [46]. Under the conditions that were employed (i.e. 500 µg.ml^−1^ tryptophan added to the growth medium), all bacterial isolates produced IAA, indicating that IAA production is common in *B. xylophilus* associated bacteria. This suggests a possible role of bacteria associated to the nematode in the PWD by suppressing the HR of pine trees.

The bacteria resistance to antibiotics observed here represents an important ecological factor in the evolution of the nematode microbial communities. Among the 35 isolates, seven isolates from symptomatic trees were resistant to all the antibiotics tested (Kan, aminoglycosides; Amp, β-lactams; Ery, macrolides; Tet, Tetracycline; and Rif, Rifamicins) suggesting bacterial fitness to subsist and proliferate inside the tree environment, and to resist chemical bactericides possibly used as disease control agents. Moreover, this feature may be transferred to other surrounding bacterial populations by horizontal gene transfer (HGT) mechanisms [47].

Both ACC deaminase production and phosphate solubilization processes have been shown to be important mechanisms in the plant growth promoting abilities of many bacteria isolated from various plant hosts [48]. Interestingly, ACC deaminase activity and the ability to solubilize phosphate were nonexistent features in the 35 bacterial isolates that were examined, suggesting that plant growth promotion by these mechanisms is not common trait in *B. xylophilus* associated bacteria. This is consistent with the results obtained in the canola root elongation assay, where it was found that only 4 of 35 isolates were able to significantly increase canola root length, even though these bacteria were poor IAA- producers. Furthermore, it was found that 11 of the 35 isolates significantly inhibited canola root elongation. The remaining 20 isolates had no significant effect on canola root development.

The pathogenicity tests of bacteria associated with *B. xylophilus* were tested in 1 year old clone trees of maritime pine, *Pinus pinaster*. The use of clone trees allowed the reduction of the genotypic variation influence in the pine response to bacteria, which was not been considered in other studies [7], [8]. Han et al. [7] reported pine wilting after inoculation (5 days) with three bacteria (*Pseudomonas fluorescens* biotype I, *P. fluorescens* biotype II and *Pantoea* sp.) in 2 month old seedlings of *P. thunbergii*. By contrast, Zhao and co-workers [8] showed no wilting in 4 month old *P. thunbergii* seedlings (five days after inoculation) by inoculation with *P. fluorescens*, *P. cepacia*, *P. putida*, and *Pantoea sp.*, nor in 3 years old *P. thunbergii* trees (3 months after inoculation) inoculated by *P. fluorescens*. More recently, Zhu et al. [49] showed that inoculation of aseptic microcuttings and seedlings of *P. densiflora* and *P. massoniana* with two bacterial isolates (*Rhizobium* sp. and *Pseudomonas* sp.) isolated from *B. xylophilus* did not wilt after 20 days. For the first time, this work reports the ability of different bacterial species associated to *B. xylophilus* (*Serratia* sp., *Enterobacter* sp. and *Pantoea* sp.) to induce similar PWD symptoms in *P. pinaster*, 45 days after inoculation.

The microbial community from *B. xylophilus* may represent a structured and cooperative multi-species consortium which, depending on the migration stage of the nematode within the host tree, may express niche-specific traits that facilitate their growth and also augment the deleterious effects of *B. xylophilus*. It has been suggested that *B. xylophilus* may be associated with ectosymbiotic bacteria living in and subsisting nutritiously from the body-surface layer of this nematode [50]. The work presented in this manuscript shows the phenotypic plasticity of *B. xylophilus* associated bacteria with plant pathogen characteristics such as moderate to high EPS- and siderophores production, with detectable cellulolytic activity and antibiotic resistance features. Furthermore, the isolates tested in *P. pinaster* seedlings can induce some PWD symptoms supporting the possibility that these bacteria play a role in *B. xylophilus* pathogenicity.
